# SOCIAL CONNECTIONS AND SUICIDE DESIRE AMONG COMMUNITY-DWELLING OLDER ADULTS

**DOI:** 10.1093/geroni/igad104.2194

**Published:** 2023-12-21

**Authors:** Mary Chase Mize

**Affiliations:** Agnes Scott College, Atlanta, Georgia, United States

## Abstract

As the population of older adults in the U.S. continues to increase, so will the prevalence of critical mental health concerns such as suicide (CDC, 2020). The Interpersonal Theory of Suicide posits desire for suicide develops from the simultaneous presence of thwarted belongingness and perceived burdensomeness (Joiner, 2005). Low social connection and perceptions of low social support can result in feelings of thwarted belongingness and perceived burdensomeness among older adults (Van Orden et al., 2012). The purpose of this study was to understand the relationship between social connections (frequency of contact with others, both synchronous or “real-time” and/or asynchronous) and suicide desire among community-dwelling older adults who received home-delivered meals (HDM) during Covid-19. This cross-sectional study was part of a grant-funded project by the Administration for Community Living. Among a sample of 320 older adults in a large southeast metropolitan area, we identified 25 participants who had no social connections of any type within the past week. These individuals had the highest levels of suicide desire and loneliness, and had the lowest levels of perceived social support. Implications for practice and future research include: a) recognition of the social connections HDM volunteers provide to older adults who may be lonely and at risk of suicide, b) an understanding of suicide desire among older adults during the Covid-19 pandemic, and c) opportunities for nutrition service providers to invest in equipping HDM volunteers to respond to suicide.

